# Self-Prioritization Effect in Children and Adults

**DOI:** 10.3389/fpsyg.2022.726230

**Published:** 2022-06-16

**Authors:** Divita Singh, Harish Karnick

**Affiliations:** ^1^School of Arts and Sciences, Ahmedabad University, Ahmedabad, India; ^2^Department of Cognitive Science, Indian Institute of Technology Kanpur, Kanpur, India

**Keywords:** self-bias, associative learning, perceptual matching, cognitive-developmental changes, perceptual saliency

## Abstract

Self-related information is processed with priority, an effect known as the self-prioritization effect (SPE). Recent studies on SPE show enhanced cognitive processing of the newly learned self-association compared to non-self (such as mother, friend, and stranger) associations among younger and older adults. However, developmental influences on the magnitude of SPE remain poorly understood. In order to examine the developmental impacts on the SPE, in the present study, we recruited participants ranging from 9–22 years of age and divided them into three age groups: older children (age 9–13), teenagers (age 14–17), and young adult (age 18–22) and compared their performance in the matching judgment task. Our results show more significant bias toward self than mother, friend, or stranger condition in all the three age groups, showing robust SPE in the 9-22-year-old age group. We also observed a more significant bias toward mother-association than friend and stranger-association in all the age groups showing an enhanced bias toward mother. Our study extends the SPE in older children and teenagers and shows that SPE remains robust and stable throughout childhood.

## Introduction

Over the years, research on “self” has shown that self-related or self-associated information has a significant advantage in cognitive processing over the information that is not related to one’s self (Brédart et al., 2006; Devue and Brédart, 2008; Turk et al., 2011; Alexopoulos et al., 2012; Zhao et al., 2015; Brédart, 2016; Oakes and Onyper, 2017). Recently, Sui et al. (2012) showed the self-advantage in processing newly learned self-association using a newly developed *perceptual matching task.* Specifically, Sui et al. (2012) showed faster responses for the newly learned geometric shapes associated with self but not for the shapes associated with the friend or stranger. In a standard matching task, participants first (at the training phase) learn the pairing of the geometric shapes (such as triangle, circle, and square) with the three labels (such as over self, friend, and stranger). Later, in the experimental phase, participants are asked to judge whether the briefly presented shape-label pairs are congruent or incongruent with respect to the pairings learned in the training phase. The presented shape-label pairing could be either congruent (i.e., match condition) or incongruent (i.e., non-match condition). Participants are overall faster in the matching condition compared to that in the non-matching condition. Interestingly, faster reaction time (RT) was reported in the self-associated shape-label matching condition compared to friend- and stranger-associated shape-label matching condition, showing a cognitive advantage for the newly learned self-associated geometric shapes over other associations (such as friend and stranger; Sui et al., 2012). Additionally, the cognitive benefit for self-associated shapes persisted even when participants made matching judgment on self and mother-associated pairs (Experiment 2 of Sui et al., 2012), suggesting that self-referential benefit is particular to the self and is not shared even with those close to self (such as mother; however see Verma et al., 2021 for cultural influences on mother-bias). This cognitive benefit for self is proposed to be driven by the heightened perceptual saliency of the self-associated items. That is, associating self with a neutral object increases that object’s overall saliency, leading to enhanced cognitive processing, similar to a perceptually salient stimulus (Humphreys and Sui, 2015; Sui and Humphreys, 2015; Sui et al., 2015; however see also Siebold et al., 2015; Stein et al., 2016; Noel et al., 2017; Reuther and Chakravarthi, 2017; Wade and Vickery, 2017, 2018; Janczyk et al., 2019; Woźniak and Hohwy, 2020).

Utilizing the approach given by Sui et al. (2012), recent empirical investigations on self have shown a self-referential advantage on many facets of cognition, such as self-advantage in attention (Sui et al., 2009; Sui and Humphreys, 2017b; Macrae et al., 2018; Wade and Vickery, 2018; Sel et al., 2019), self-advantage in action (Sui and Humphreys, 2017b; Desebrock et al., 2018; Dalmaso et al., 2019; however, see also Nijhof et al., 2020), and greater distortion in time estimation by self-association (Makwana and Srinivasan, 2019). The self-referential effect has been observed in audition and touch (Schäfer et al., 2016), reinforcing the idea of common information processing of self across various senses. Additionally, self-advantage has been found only for the objects associated with the current self and not for the objects associated with the past and future self (Golubickis et al., 2017). Moreover, the self-reference effect has been observed cross-culturally (Jiang et al., 2019), suggesting that the self-bias does not depend on the kind of society one belongs to (such as individualistic society or collective society; however, see Verma et al., 2021). For example, Jiang et al. (2019) showed a comparable self-advantage in the participants from the UK as well as Hong Kong (HK), suggesting that self-bias could be a universal phenomenon (however, HK participants show similar performance in the friend and stranger category; also see Zhu and Han, 2008). Furthermore, Maire et al. (2020) recently reported SPE in younger children (6–10-year-old children), suggesting that SPE is a strong effect that starts very early. Taken together, recent literature suggests a vital role of newly acquired self-association on information processing wherein self-associated information is prioritized over that of a friend-, mother-, and stranger-associated information. Nevertheless, this prioritization of self over mother and friend demonstrates that self receives more weightage in processing than friend and mother.

Despite the extensive work concerning SPE and various cognitive processes, less attention has been paid to understanding developmental influences on the SPE. The reason why developmental studies on SPE are required is that (1) self is a phenomenon that seems to be highly influenced by the developmental changes (Harter, 1988; Nobre and Valentini, 2019), and (2) conceptualization of one’s self changes extensively in the teen years (Steinberg, 2005; Pfeifer et al., 2007; Sebastian et al., 2008). Besides, examining the developmental influences on SPE and comparing the extent of the bias for self, mother, and friend will also help broaden our understanding of SPE.^1^ Thus, the present study aimed to examine developmental influences on the cognitive processing of self-associated information, mother-associated information, and friend-associated information in the matching judgment task. Developmental studies have shown that the teen years are crucial as concentration on oneself gradually changes in these years (Herba and Phillips, 2004; Yurgelun-Todd and Killgore, 2006). On the contrary, developmental studies show that attachment to the mother seems to be greatest during age 6–10 years (Buist et al., 2002; Ruhl et al., 2015). Hence, age-related changes in the orientation toward self and mother might influence the magnitude of association strength of the shape and label.

With this aim, we recruited participants ranging from the age of 9–22 years and categorized them into three age groups^2^: older children (age 9–13), teenagers (age 14–17), and young adults (age 18–22) and included four label conditions: self, mother, friend, and stranger. Recent studies on Self-prioritization have shown slight differences in the prioritization of different labels and degrees to which each of them differs from self. For example, some studies show comparable responses in friend and stranger conditions (Zhu and Han, 2008), and some show comparable responses in self and mother (Verma et al., 2021). Thus, in order to capture these variations, we decided to include all four labels. We expect a stronger SPE in all three age groups and developmental influences over the magnitude of SPE (if any). Additionally, we aim to analyze the within age group effects to examine the cognitive bias for each association in each group separately. Looking at both between and the within-group results will provide developmental influences on the magnitude of SPE and association effects in each age group.

## Materials and Methods

### Participants

The study had a total of 52 participants [17 participants in the older children group (7 females and 8 males; average age = 11), 19 participants in the teenage group (9 females, 10 males; average age = 15.5), and 16 participants in the young adult’s group (8 females, 8 males; average age = 20)]. All the participants reported normal or corrected to normal vision and were right-handed. All of them gave informed consent before the commencement of the experimental session. Parental consent was obtained in the case of older children and teenagers. The protocol was approved by the Indian Institutional Technology Institutional Ethics Committee. The sample size was decided from an *a priori* power analysis conducted using G*Power3 (Faul et al., 2007) with the power (1 − β) of 0.95, the effect size of (*η*^2^) = 0.41 (based on Sui et al., 2012), and alpha of 0.05.

### Apparatus and Stimulus

Participants were seated in a dimly lit room in front of the IBM PC-compatible computer with a 19-inch LG LED monitor at a 1920 Χ 1080 pixel resolution. The experiment was created using PsychoPy3 Experiment builder software (Peirce, 2007, 2009). Participants’ responses were recorded using the “Z” and “M” keys on the standard keyboard. The stimuli used in the experiment were black-colored geometric shapes presented on a white background.

### Procedure and Design

**Figure 1:**
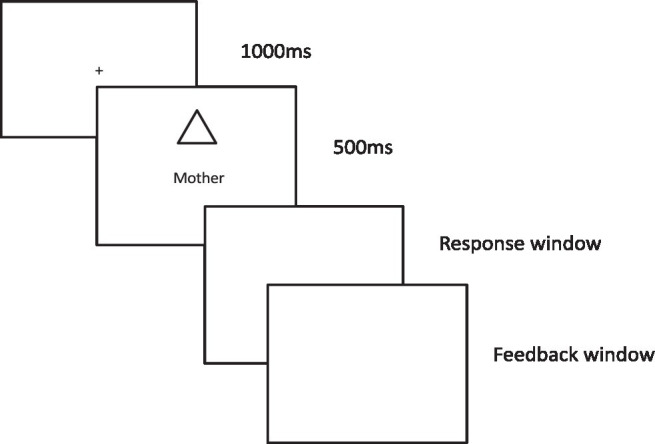
An illustration of the experiment trial with the mother as a label and a triangle as an associated shape. The participant’s task was to report whether the presented shape-label pairing was correct or incorrect.

Upon arrival at the laboratory, participants were provided with the basic information about the task, and then informed consent was taken. After the general familiarization with the laboratory setting, participants were asked to recall the name of their best friend. Once they had told the name of their best friend, they were instructed to close their eyes and listen to the instructor carefully. Participants were then required to memorize the following information: Girish (participant’s name, for example) you have to remember that you are a triangle, your mother is a rectangle, Shaskank (participant’s best friend’s name as reported by the participant) is square, and Raghu (an unknown person to the participant) is a circle. After giving this information, participants were given a few minutes (maximum 5 mins) to memorize this association (shape and their label). Shape and labels were not shown at this stage. Once the participant was ready and had memorised the newly formed associations, they were shown each shape and label on the computer screen. At the same time, the instructor tried to probe them by asking whether the displayed shape-label pairing was correct or incorrect (as communicated during the instruction). After showing the shape and label pairs, participants completed 15 practice trials. Each trial started with the presentation of a fixation cross on the center of the screen for 1,000 ms, followed by the shape and the label for 500 ms (see Figure 1). After the presentation of the shape and label, the response window appeared, which remained open for 3,000 ms. Participants were encouraged to respond as soon as possible. After each response, feedback “correct” or “incorrect” was provided. The feedback was displayed for 1,000 ms. The next trial started immediately after the feedback. The shape appeared at 3.8° × 3.8° above the fixation cross, and the label appeared at 3.6° × 1.6° below the fixation cross. Shape and label pairings were counterbalanced across the participants. Participants pressed the “M” key when the presented shape and label matched and the “Z” key for the non-matched shape-label pairings. To avoid any response bias, response keys were counterbalanced across the participants. After the practice trial, participants proceeded to the full experiment.

The experiment had 416 trials (with 52 trials in each condition), divided into two blocks. A forced short break of 2 min was inserted after the first block. After the 2-min break, a window appeared asking participants to press any key to resume the experiment. Participants were allowed to take a break of more than 2 min and pressed the key whenever ready. The whole experiment lasted for about 40–45 min. After completing the experiment, participants were debriefed about the purpose of the experiment and were allowed to ask any questions that they might have regarding the experiment.

## Results

Following the previous literature on self-bias, match and mismatch trials were analyzed separately as they reflect different response criteria (Sui et al., 2012; Sui and Humphreys, 2017a). Two participants from the older children group were excluded from the final analysis due to the very high error rate (80%). In addition, trials shorter than 100 ms and longer than 3,000 ms were excluded from the final analysis, leading to the removal of 18 trials in total from the final analysis.

### Match Trials

**Figure 2:**
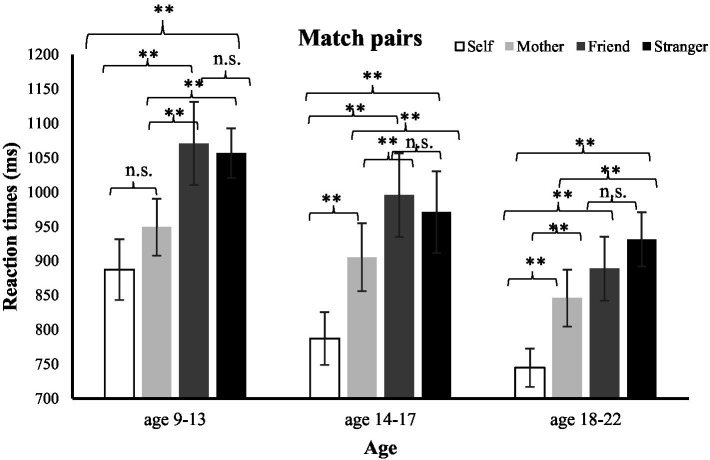
Mean RTs in the matching judgment of the matched shape-label pairs as a function of age (9–13, 14–17, and 18–22) and association (self, mother, friend, and stranger). Error bars show standard error. ^**^significant; n.s. not significant.

Repeated-measure ANOVA was conducted with the shape-label association (self, mother, friend, and stranger) as a within-subject factor and age [older children (9–13), teenagers (14–17), and young adults (18–22)] as a between-subject factor (see Figure 2) on RT as well as accuracy.

#### Accuracy

A 3 (age groups: older children, teenagers, and young adults) × 4 (labels: self, mother, friend, and stranger) repeated-measure ANOVA computed on RT data showed a main effect of association *F*(3, 141) = 35.456, *p* < 0.001, $\eta_{p}^{2}$ = 0.43, with faster responses in self (mean RT = 806 ms)- and mother-association condition (mean RT = 899 ms) than friend (mean RT = 984 ms)- and stranger-association condition (mean RT = 986). Post-hoc comparison showed that all the associations were significantly different from each other (*p* < 0.001), except friend- and stranger-association condition (*p* = 1.0). The result also showed a marginal main effect of age, *F*(2, 47) = 2.47, *p* = 0.09, $\eta_{p}^{2}$ = 0.10, suggesting that older participants were overall faster than the younger participants. Further, a planned post-hoc analysis (Bonferroni correction method) showed that the marginal effect of age was due to the significant difference between the RT of the older children group and young adult group (*p* < 0.05), suggesting that the older children group were overall slower (mean RT = 990 ms) in the matching task than the young adult group (mean RT = 852 ms). However, older children and the teenage group were not significantly different (*p* = 0.24), suggesting that the teenage group’s overall response rate (mean RT = 914 ms) was comparable with the older children group. Also, the overall response rate of teenage and young adult groups was not significantly different (*p* = 0.32). Moreover. the interaction between age and association was not significant *F*(6, 141) = 0.709, *p* = 0.643, $\eta_{p}^{2}$ = 0.03.

#### RT

A 3 (age groups: older children, teenagers, and young adults) × 4 (labels: self, mother, friend, and stranger) repeated-measure ANOVA computed on accuracy data showed a main effect of association *F*(3, 141) = 23.45, *p* < 0.001, $\eta_{p}^{2}$ = 0.32, with greatest accuracy in self (mean accuracy = 94.5%)- and mother-association (mean accuracy = 90.12%) and lowest in friend (mean accuracy = 86.72%)- and stranger-association condition (mean accuracy = 83.61%). Results also showed a significant main effect of age, *F*(2, 47) = 3.66, *p* < 0.05, $\eta_{p}^{2}$ = 0.13, showing greatest accuracy in young adults (mean accuracy = 92.5%) and lowest in older children (mean accuracy = 89.8%) and teenage group (mean accuracy = 84.4%). Moreover, the interaction between age and association was not insignificant (*p* = 3).

As we also wanted to examine the within-group effects for each association, within-subject ANOVA was conducted separately for each age group. In addition, a paired-samples *t*-test (Student’s *t*-test) and Bayesian paired samples *t*-test were performed on each possible association pair to analyze the within-group effects.

Repeated-measure ANOVA conducted on the older children showed a main effect of association, *F*(3, 41) = 8.75, *p* < 0.001, $\eta_{p}^{2}$ = 0.39, with faster reaction time to the self-compared to both friend-associated trials [*t*(14) = −4.49, *p* < 0.001, *dz* = 1.16; BF_10_ = 68.9] and stranger-associated trials [*t*(14) = −4.64; *p* < 0.001; BF_10_ = 84.4]. Similarly, faster response was observed in the mother-associated trials compared to both friend [*t*(14) = −2.66, *p* < 0.05, *dz* = 0.69; BF_10_ = 3.32] and stranger-associated trials [*t*(14) = −2.90, *p* < 0.05, *dz* = 0.75; BF_10_ = 4.89]. Most importantly, responses in the self and mother-associated trials did not differ significantly from each other [*t*(14) = −1.62, *p* = 0.13, *dz* = 0.42; BF_01_ = 1.32]. We also found no significant difference in the responses between friend and stranger-associated trials [*t*(14) = 0.25, *p* = 0.8, *dz* = 0.06; BF_01_ = 3.70].

Analysis on the teenage group also showed a significant effect of association, *F*(3, 54) = 14.86, *p* < 0.001, $\eta_{p}^{2}$ = 0.45. However, unlike the older children group, teenage group showed faster responses in the self-associated trials than mother [*t*(18) = −4.20, *p* < 0.001, *dz* = 0.96; BF_10_ = 63.8]-, friend [*t*(18) = −5.20, *p* < 0.001, *dz* = 1.19; BF_10_ = 435.2]-, and stranger-associated trials [*t*(18) = 4.55, *p* < 0.001, *dz* = 1.04; BF_10_ = 124.3]. Furthermore, mother-associated trials were significantly different from friend [*t*(18) = −3.6, *p* < 0.05, *dz* = 0.83; BF_10_ = 20.6] but not stranger-associated trials [*t*(18) = −1.7, *p* < 0.05, *dz* = 0.40; BF_01_ = 1.15]. Additionally, there was no significant differences between friend- and stranger-associated trials [*t*(18) = 0.78, *p* = 0.44, *dz* = 0.18; BF_01_ = 3.19].

Significant effect of association was also obtained in the young adult group, *F*(2, 102423.37) = 17.35, *p* < 0.001, $\eta_{p}^{2}$ = 0.54, with faster responses in the self-associated trials than mother [*t*(15) = −3.47, *p* < 0.005, *dz* = 0.87; BF_10_ = 13.28]-, friend [*t*(15) = −4.40, *p* < 0.001, *dz* = 1.10; BF_10_ = 67.04]-, and stranger-associated trials [*t*(15) = −8.12, *p* < 0.001, *dz* = 2.03; 23,650]. However, responses did not differ between the friend- and stranger-associated trials [*t*(15) = −1.39, *p* = 0.18, *dz* = 0.35; BF_01_ = 1.72]. Moreover, responses in the mother-associated trials were significantly different from friend [*t*(15) = −2.12, *p* = 0.05, *dz* = 0.53; BF_10_ = 1.5] and stranger-associated trials [*t*(15) = −3.38, *p* < 0.05, *dz* = 0.85; BF_10_ = 11.4].

### Mis-match Trials Analysis

Similar to match trials, we conducted repeated-measure ANOVA on accuracy data as well as RT data on the mis-match trials. Accuracy analysis on mis-match trials with age as a between-subject factor and shape-label association as a within-subject factor showed significant main effect of association, *F*(3, 141) = 4.63, *p* < 0.05, $\eta_{p}^{2}$ = 0.08, and age *F*(2, 47) = 3.97, *p* < 0.05, $\eta_{p}^{2}$ = 0.14, showing overall greater mean accuracy in the young adult group (self = 90.99%; mother = 91.11%, friend = 87.98%; stranger = 87.14%) and lowest mean accuracy in older children group (self = 85.52%; mother = 86.53%; friend = 81.78%; stranger = 88%) and teenage group (self = 78.85%; mother = 82.99%; friend = 77.64%; and stranger = 80.67%). Moreover, the interaction between age and association was not significant *F*(6, 141) = 0.709, *p* = 0.63, $\eta_{p}^{2}$ = 0.02.

**Figure 3:**
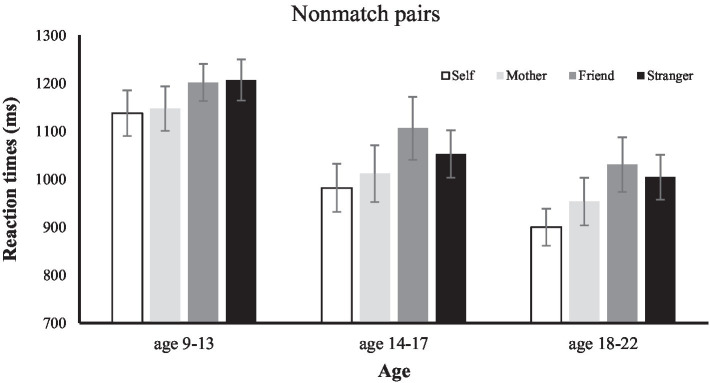
Mean RTs in the matching judgment of the non-matching shape-label pairs as a function of age (9–13, 14–17, and 18–22) and association (self, mother, friend, and stranger). Error bars show standard error.

RT analysis performed on mismatch trials with age as a between-subject factor and shape-label association as a within-subject factor showed significant main effect of association (see Figure 3), *F*(3, 141) = 20.75, *p* < 0.001, $\eta_{p}^{2}$ = 0.30, and age *F*(2, 47) = 4.10, *p* < 0.05, $\eta_{p}^{2}$ = 0.14, with faster responses in the self-mis-match trials (mean RT = 1002.26) and mother-mis-match trials (mean RT = 1033.62) compared to friend (mean RT = 1110.48) and stranger mis-match trials (mean RT =1,084). We also obtained a main effect of age, *F*(2, 47) = 4.10, *p* < 0.05, $\eta_{p}^{2}$ = 0.15. Post-hoc comparison showed that the main effect of age was due to the significant difference between the responses of older children group and the young adult group (*p* < 0.05), whereas teenage and young adult group did not show any difference in the responses of the mis-match trials (*p* = 0.34). Response of the older children and the teenagers also did not differ significantly from each other (*p* = 0.11). The interaction between age and association was not significant *F*(6, 141) = 0.87, *p* = 0.51, $\eta_{p}^{2}$ = 0.03.

#### D Primes

In order to compute the sensitivity of the discrimination, we calculated d prime scores for each age group and each association (see Table 1). The d prime was calculated by computing the sensitivity for match and mis-match trials using the Green and Swets (1966) formula: d’ = z(Hit) – z(FA). A 4 (associations: self, mother, friend, and stranger) × 4 (age groups: older children, teenagers, and young adults) repeated-measures ANOVA was then performed. Analysis showed significant main effect of association, *F*(3, 141) = 12.41, *p* < 0.001, $\eta_{p}^{2}$ = 0.21, with larger d’ for self (mean *d*’ = 1.06), than mother (mean *d*’ = 0.84), friend (mean *d*’ = 0.74), and stranger (mean *d*’ = 0.63). Results also showed a significant main effect of age, *F*(2, 47) = 5.5, *p* < 0.05, $\eta_{p}^{2}$ = 0.19. Post-hoc analysis showed that the main effect of age was driven by the significant difference between teenagers and young adults (*p* < 0.05). All the other pairs were non-significant.

**Table 1:** Mean D′ scores for each association and age groups.

	Self	Mother	Friend	Stranger
Age 9–13	1.128	0.787	0.779	0.632
Age 14–17	0.87	0.655	0.632	543
Age 18–22	1.29	1.11	1.01	0.887

## Discussion

The current study sought to study the developmental influences on the cognitive processing of newly learned associations in the 9–22 years’ age group. To examine the developmental influences on the associations, performance in the matching judgment of shape-label matched pairs was compared across age. Results showed cognitive benefit for self-association in all three age groups, showing a robust SPE between 9 and 22 years’ age group. With this, we not only replicated the standard SPE as observed in the previous literature (Sui et al., 2012; Oakes and Onyper, 2017; Yankouskaya et al., 2017; Kim et al., 2019; Woźniak and Knoblich, 2019) but also extended the SPE in the older children and teenagers and showed that SPE remains robust across the developmental years. The observed bias for the newly learned self-association in the older children group is consistent with the previous work on SPE in younger children (6–10-year-old; Maire et al., 2020) and self-ownership studies, which show greater memory (Cunningham et al., 2013, 2014) and greater retention rate for the self-referent objects compared to others-referent objects in the early childhood years (3–6 year old; Axelsson et al., 2018). Our result also showed an overall decrease in the matching judgment response time with age, suggesting that 9–13-year-old children were, in-general, slower than teenagers and young adults. A slower response in the children group was expected as they lack familiarity with the task setup (computer and the keyboards).

Slower response time in the older children group could have been due to the differences in the deployment of differential memory strategies and attentional control while learning the association between the two pairs. For example, developmental research has shown that adults are far more superior and fine-tuned with the deployment of attentional control in the task than young children (Beuhring and Kee, 1987; Guttentag, 1995; Luna, 2009; Schneider and Pressley, 2013). Alternatively, it is also possible that older children might have processed both self and mother-associations in equal priority (which was also evident in the greater mother processing effect (MPE) in the older children group compared to teenage and young adults). Therefore, this equal prioritization might have added extra cost in the cognitive processing, resulting in the greater RT for self and leading to the emergence of the mother bias. Equal prioritization of self- and mother-association holds greater weight as 9–13-year-old children are considered closer with their mother than teenagers and young adults. For example, developmental studies have shown that children of age 6–13 years are very attached to their mother (Buist et al., 2002; Ruhl et al., 2015), and this attachment tends to decrease once they reach teenage (age 14–17; Steinberg, 2001; Smetana et al., 2006; Van Doorn et al., 2008; Asher et al., 2020). This personal closeness (and thus familiarity) with the mother might have influenced the strength of mother-association and thus modulated the perceived saliency of shape-label associations. This proposal is also in line with the previous literature on SPE, which suggest that familiarity (Sui and Humphreys, 2017b) and easily imaginable labels (Wade and Vickery, 2017) modulate the magnitude of bias by generating a stronger association.

Moreover, it is undeniable that cognitive developmental changes are very rapid and significant in the teenage years (Herba and Phillips, 2004; Blakemore and Choudhury, 2006; Choudhury et al., 2006; Yurgelun-Todd and Killgore, 2006) and might have influenced the perceived familiarity and thus association acquisition. For example, from the age of 13–14 years (teenager), children tend to become more independent, and during this period, relationship with their parents (specifically the mother) changes at a great length (Laursen et al., 1998, 2010; Karabanova and Poskrebysheva, 2013; Branje, 2018). The conflicts between parent and child increase as they move into the teenage (Noller and Callan, 1986; Larson et al., 1996; Steinberg, 2001; Steinberg and Morris, 2001; Allen et al., 2004; Smetana et al., 2006; Van Doorn et al., 2008; De Goede et al., 2009). In the teenage years’ gradual focus toward ‘self’ starts, and children become more self-conscious (Steinberg, 2005). This conflict with parents and increased self-consciousness might add to the personal distance between self and mother in the teenage, affecting the strength and priority of self- and mother-associations. Since we did not use any measure to assess the personal closeness prior to the matching task, we cannot directly predict the role of personal distance and strength of self and mother bias observed in our study. Future studies should utilize the personal distance measures and examine the correlation between SPE, MPE, and personal distance score to study the influence of the parent–child relationship and parent–child attachment on self-prioritization and associative learning to assess the direct link. The small sample size is another limitation of this study. Although the chosen sample size goes along with the sample sizes used in the SPE studies, a larger sample size could have generated more significant results and would have greater power. Additionally, in hindsight, we feel that more extensive practice trials for the young children group could have helped minimize the task setup familiarity effect and slower responses, making the results clearer.

## Conclusion

Taken together, our study reports SPE and MPE in the 9–22 age group and show that self-prioritization remains stable until the age of young adulthood. Most importantly, our study suggests that cognitive-developmental changes during the developmental years (i.e., between age 10–17) do not influence the associative learning of self and mother labels in the perceptual matching task. Despite extensive changes in the conceptualization of “self” during the teen years, the strength of self-prioritization does not differ significantly from older children to young adulthood.

## Data Availability Statement

The raw data supporting the conclusions of this article will be made available by the authors, upon personal request.

## Ethics Statement

The studies involving human participants were reviewed and approved by the IIT Kanpur Institutional Ethics Committee. Written informed consent to participate in this study was provided by the participants’ legal guardian/next of kin.

## Author Contributions

DS: conceptualization, methodology, formal analysis, investigation, writing—original draft, and visualization. HK: supervision, conceptualization, and methodology. Both authors contributed to the article and approved the submitted version.

## Funding

The reported study was funded by the Department of Science and Technology—Cognitive Science Research Initiative Post-Doctoral Fellowship research fund to DS (Project Grant No: DST/CS/2018133).

## Conflict of Interest

The authors declare that the research was conducted in the absence of any commercial or financial relationships that could be construed as a potential conflict of interest.

## Publisher’s Note

All claims expressed in this article are solely those of the authors and do not necessarily represent those of their affiliated organizations, or those of the publisher, the editors and the reviewers. Any product that may be evaluated in this article, or claim that may be made by its manufacturer, is not guaranteed or endorsed by the publisher.

## Footnotes

^1^Most of the research on SPE employs minimal self rather than narrative self. Future research is needed to see how SPE pans out in the context of narrative self.

^2^Older children age range was based on Burnett Heyes et al. (2015), and teenagers age range was based on Vuontela et al. (2003).
